# *PIK3R1* underexpression is an independent prognostic marker in breast cancer

**DOI:** 10.1186/1471-2407-13-545

**Published:** 2013-11-14

**Authors:** Magdalena Cizkova, Sophie Vacher, Didier Meseure, Martine Trassard, Aurélie Susini, Dana Mlcuchova, Celine Callens, Etienne Rouleau, Frederique Spyratos, Rosette Lidereau, Ivan Bièche

**Affiliations:** 1 Oncogenetic Laboratory, Institut Curie, Hospital René Huguenin, Saint-Cloud, France; 2 Department of Oncology, Faculty of Medicine and Dentistry, Palacky University and University Hospital Olomouc, Olomouc, Czech Republic; 3 Laboratory of Experimental Medicine, Institute of Molecular and Translational Medicine, Faculty of Medicine and Dentistry, Palacky University and University Hospital Olomouc, Olomouc, Czech Republic; 4 Department of Pathology, Institut Curie, Hospital René Huguenin, Saint-Cloud, France; 5 UMR745 INSERM, Université Paris Descartes, Sorbonne Paris Cité, Faculté des Sciences Pharmaceutiques et Biologiques, Paris, France; 6 Laboratoire d’Oncogénétique, 35 rue Dailly, Institut Curie - Hôpital René Huguenin, St-Cloud F-92210, France

**Keywords:** *PIK3R1*, *PIK3CA*, Breast cancer, Breast cancer subtypes, Signaling pathways, Prognostic value

## Abstract

**Background:**

The present study focused on the prognostic roles of *PIK3CA* and *PIK3R1* genes and additional PI3K pathway-associated genes in breast cancer.

**Methods:**

The mutational and mRNA expression status of *PIK3CA, PIK3R1* and *AKT1*, and expression status of other genes involved in the PI3K pathway (*EGFR*, *PDK1*, *PTEN, AKT2, AKT3, GOLPH3, WEE1, P70S6K)* were assessed in a series of 458 breast cancer samples.

**Results:**

*PIK3CA* mutations were identified in 151 samples (33.0%) in exons 1, 2, 9 and 20. *PIK3R1* mutations were found in 10 samples (2.2%) and underexpression in 283 samples (61.8%). *AKT1* mutations were found in 15 samples (3.3%) and overexpression in 116 samples (25.3%). *PIK3R1* underexpression tended to mutual exclusivity with *PIK3CA* mutations (p = 0.00097). *PIK3CA* mutations were associated with better metastasis-free survival and *PIK3R1* underexpression was associated with poorer metastasis-free survival (p = 0.014 and p = 0.00028, respectively). By combining *PIK3CA* mutation and *PIK3R1* expression status, four prognostic groups were identified with significantly different metastasis-free survival (p = 0.00046). On Cox multivariate regression analysis, the prognostic significance of *PIK3R1* underexpression was confirmed in the total population (p = 0.0013) and in breast cancer subgroups.

**Conclusions:**

*PIK3CA* mutations and *PIK3R1* underexpression show opposite effects on patient outcome and could become useful prognostic and predictive factors in breast cancer.

## Background

The phosphatidylinositol 3-kinase (PI3K) pathway has been identified as an important player in cancer development and progression. Following receptor tyrosine kinase activation, PI3K kinase phosphorylates inositol lipids to phosphatidylinositol-3,4,5-trisphosphate. The level of phosphatidylinositol-3,4,5-trisphosphate is regulated by phosphatase activity of PTEN. Signal transmission subsequently leads to PDK1 followed by activation of AKT. AKT then regulates activation of the pathway downstream effectors, including mTOR and subsequently P70S6K as well as other targets such as GSK3, WEE1 or BAD. mTOR has been found to be positively regulated by GOLPH3. The PI3K pathway controls important cellular processes such as protein synthesis, cell growth and proliferation, angiogenesis, cell cycle and survival [1-3].

PI3K pathway deregulation is frequent in tumor cells and can be caused by multiple changes affecting different levels of the signaling cascade. These changes include gene amplifications, mutations and expression alterations. However, various patterns of PI3K pathway changes have been identified in different cancer types. In breast cancer, such events commonly affect receptor tyrosine kinases, *PTEN*, *PIK3CA* and, to a lesser degree, *AKT1*. *PIK3CA* as well as *AKT1* mutations have been described as early events in the breast cancer development process [3-6].

PI3K is a heterodimer and consists of a p110α catalytic subunit encoded by the *PIK3CA* gene and a p85 regulatory subunit alpha encoded by the *PIK3R1* gene [7-11]. The *PIK3CA* oncogene is a well known site of activating hot spot mutations located in exons 9 and 20, corresponding to the helical (E542K and E545K) and kinase (H1047R) domains, respectively. *PIK3CA* mutations are among the most common mutations, as they are observed in 10 to 40% of breast cancer cases, depending on the breast cancer subtype [3,4,8,12]. *PIK3CA* carrying a hotspot mutation exerts an oncogenic activity: it can transform primary fibroblasts in culture, induce anchorage-independent growth, and cause tumors in animals [13,14]. Apart from exons 9 and 20, *PIK3CA* has been recently shown to be also mutated frequently in other exons, as demonstrated by Cheung *et al.* in the case of endometrial cancer [15]. On the contrary, the *PIK3R1* gene appears to play a tumor suppressor role because PI3K subunit p85α (p85α) regulates and stabilizes p110α [7,16]. *PIK3R1* has also been recently found to be mutated in breast cancer, but with a considerably lower frequency (about 3%) than *PIK3CA*[17]. The impact of its suppressor activity needs to be further described in breast cancer. It is noteworthy that other PI3K subunit encoding genes (*PIK3CB*, *PIK3CD*, *PIK3CG*, *PIK3R2*, *PIK3R3*) are altered with much lower frequency than *PIK3CA* and *PIK3R1*[17]. Loss of PTEN expression, observed in about 20-30% of cases, is known to be one of the most common tumor changes leading to PI3K pathway activation in breast cancer [4].

Discordant reports have been published concerning the prognostic role of *PIK3CA* mutations [4,18,19]. These mutations appear to be preferentially associated with more favorable clinicopathologic characteristics and more favorable outcome in breast cancer patients [3]. *PIK3R1* underexpression might possibly lead to PI3K pathway activation and confer tumor development and progression in humans in a similar way to that observed in a mouse model of hepatocellular cancer [16].

In the present study, we explored the two genes encoding PI3K subunits and their role in PI3K pathway deregulation and patient survival. *PIK3CA*, *PIK3R1* and *AKT1* mRNA expression levels and mutations were studied. We also assessed mRNA expression levels of other genes involved in the PI3K pathway, namely *EGFR*, *PDK1*, *PTEN*, *AKT1*, *AKT2, AKT3, GOLPH3*, *P70S6K*, and *WEE1* to elucidate the pathway deregulations associated with changed *PIK3CA* and *PIK3R1* states. PTEN and p85 protein expression were also assessed by immunohistochemistry.

## Methods

### Patients and samples

We analyzed 458 samples of unilateral invasive primary breast tumors excised from women at the Institut Curie/Hôpital René Huguenin (Saint-Cloud, France) from 1978 to 2008 (Additional file 1: Table S1) where majority of the patients were diagnosed and treated between years 1990 and 2000 (67%). All patients admitted to our institution before 2007 were informed that their tumor samples might be used for scientific purposes and they were given the opportunity to refuse the use of their samples. Since 2007, patients admitted to our institution also give their approval by signing an informed consent form. This study was approved by the local ethics committee (René Huguenin Hospital Breast Group). Patients (mean age: 61.7 years, range: 31–91) met the following criteria: primary unilateral non-metastatic breast carcinoma, with full clinical, histological and biological data; no radiotherapy or chemotherapy before surgery; and full follow-up at Institut Curie/Hôpital René Huguenin. Median follow-up was 8.6 years (range: 4.3 months to 28.9 years). One hundred and seventy patients developed metastases.

Samples were examined histologically and were considered suitable for this study when the proportion of tumor cells exceeded 70% with sufficient cellularity, as demonstrated by evaluation of tumor samples stained by hematoxylin and eosin. Immediately following surgery, tumor samples were placed in liquid nitrogen until RNA extraction and also stored as formalin-fixed paraffin-embedded tumor tissue sample blocks for immunohistochemistry analysis.

Treatment consisted of modified radical mastectomy in 283 cases (63.9%) and breast-conserving surgery plus locoregional radiotherapy in 160 cases (36.1%). None of the ERBB2-positive patients was treated by anti-ERBB2 therapy. Clinical examinations were performed every 3 or 6 months for the first 5 years according to the prognostic risk of the patients, then yearly. Mammograms were done annually. Adjuvant therapy was administered to 358 patients, consisting of chemotherapy alone in 90 cases, hormone therapy alone in 175 cases and both treatments in 93 cases. The histological type and number of positive axillary nodes were established at the time of surgery. The malignancy of infiltrating carcinomas was scored with Bloom and Richardson’s histoprognostic system.

Estrogen receptor (ER) and progesterone receptor (PR) status was determined at the protein level by using biochemical methods (dextran-coated charcoal method or enzyme immunoassay) until 1999 and then by immunohistochemistry. The cutoff for estrogen and progesterone receptor positivity was set at 15 fm/mg (dextran-coated charcoal or enzyme immunoassay) and 10% immunostained cells (immunohistochemistry). A tumor was considered ERBB2-positive by IHC when it scored 3+ with uniform intense membrane staining > 30% of invasive tumor cells. Tumors scoring 2+ were considered to be equivocal for ERBB2 protein expression and were tested by FISH for ERBB2 gene amplification. In all cases, the ERα, PR and ERBB2 status was also confirmed by real-time quantitative RT-PCR with cutoff levels based on previous studies comparing results of the these methods [20-23]. Based on HR (ERα and PR) and ERBB2 status, the 458 patients were subdivided into 4 subgroups as follows: HR- (ER- and PR-)/ERBB2- (n = 69), HR- (ER- and PR-)/ERBB2+ (n = 45), HR + (ER + or/and PR+)/ERBB2- (n = 290) and HR + (ER + or/and PR+)/ERBB2+ (n = 54).

### RNA extraction

Total RNA was extracted from breast tumor samples by using the acid-phenol guanidium method. The quantity of RNA was assessed by using an ND-1000 NanoDrop Spectrophotometer with its corresponding software (Thermo Fisher Scientific Inc., Wilmington, DE). RNA quality was determined by electrophoresis through agarose gel and staining with ethidium bromide. The 18S and 28S RNA bands were visualized under ultraviolet light. DNA contamination was quantified by using a primer pair located in an intron of the gene encoding albumin (gene *ALB*). Only samples with a cycle threshold (Ct) using these *ALB* intron primers greater than 35 were used for subsequent analysis.

### Mutation screening

*PIK3CA* mutations (exons 1, 2, 9, 20), *PIK3R1* (exons 11–15) and *AKT1* (exon 4) were detected by sequencing of cDNA fragments obtained by RT-PCR amplification. Exons to be screened in the three genes were chosen following mutational frequency described at COSMIC: Catalogue Of Somatic Mutations In Cancer (cancer.sanger.ac.uk/). Screening by high-resolution melting curve analysis was performed on *PIK3CA* exons 1 and 2, *AKT1* exon 4 and *PIK3R1* exons 11 to 15 on a LightCycler 480 (Roche Diagnostics, Penzberg, Germany) using LCGreen Plus + Melting Dye fluorescence (Biotech, Idaho Technology Inc., Salt Lake City, UT). Details of the primers and PCR conditions are available on request. The amplified products were sequenced with the BigDye Terminator kit on an ABI Prism 3130 automatic DNA sequencer (Applied Biosystems, Courtaboeuf, France) with detection sensitivity of 5% mutated cells, and the sequences were compared with the corresponding cDNA reference sequences (*PIK3CA* NM_006218, *PIK3R1* NM_181523, *AKT1* NM_005163). All detected mutations were confirmed in the second independent run of sample testing.

### Real-time quantitative RT-PCR

RT-PCR was applied to the selected genes and to *TBP* (NM_003194) as endogenous mRNA control. Primers are listed in Additional file 2: Table S2. PCR conditions are available on request. The RT-PCR protocol using the SYBR Green Master Mix kit on the ABI Prism 7900 Sequence Detection System (Perkin-Elmer Applied Biosystems, Foster City, CA) is described in detail elsewhere [20]. The relative mRNA expression level of each gene, expressed as the N-fold difference in target gene expression relative to the *TBP* gene, and termed “N*target*”, was calculated as N*target* = 2^ΔCt*sample*^. The value of the cycle threshold (ΔCt) of a given sample was determined by subtracting the average Ct value of the target gene from the average Ct value of the *TBP* gene. The N*target* values of the samples were subsequently normalized so that the median N*target* value of normal breast samples was 1. Cut-offs for normalized values ≤ 0.5 and ≥ 2.0 were used to determine gene underexpression and overexpression, respectively.

### Immunohistochemistry

PTEN and p85 protein expression levels were assessed by immunohistochemistry staining on tumor sections from formalin-fixed paraffin-embedded blocks. Indirect immunoperoxidase staining was performed using mouse monoclonal antibody directed against human PTEN protein (Dako, Glostrup, Denmark) and rabbit polyclonal antibody directed against human p85 protein (Signalway Antibody, Baltimore, Maryland). The localization and intensity of staining were assessed by two independent pathologists blinded to real-time RT–PCR results.

Both antibodies were used at a 1/50 dilution. The immunohistochemical procedure was performed as described below, using a water bath antigen-retrieval technique in each case. Sections were mounted on precoated slides (Dako, Glostrup, Denmark) and allowed to dry at 50°C overnight. Sections were then dewaxed in xylene and hydrated by graded dilutions of ethanol. Endogenous activity was blocked with 1% hydrogen peroxide for 15 min. Sections were then immersed in a heat-resistant plastic box containing 10 ml of pH 9.0 citrate buffer and processed in the water bath for 40 min. Sections were then allowed to cool to room temperature for 20 min before rinsing in H_2_O. The blocking reagent was poured off and the primary antibodies were left for 25 min. A standard avidin–biotin–peroxidase complex (LSAB) method was used to reveal the antibody–antigen reaction (Dako, Glostrup, Denmark). Autostainer link 48 was used for the staining process (Dako, Glostrup, Denmark).

Normal ductal epithelial cells showed a positive cytoplasmic immunostaining, whereas PTEN expression in tumor cells varied with cytoplasmic and/or nuclear staining. A semi-quantitative intensity score was performed (score 0: negative staining, score 1: weak cytoplasmic staining, score 2: moderate cytoplasmic staining, score 3: strong and diffuse cytoplasmic staining). Positive immunohistochemical reactions were defined as a brown cytoplasmic staining for p85. A semi-quantitative intensity scale ranging from 0 for no staining to 3+ for the most intense staining was used by comparing neoplastic cells to adjacent breast cells belonging to normal terminal ductulo-lobular units. p85 underexpression was defined by an IHC score 0, p85 normal expression by an IHC score 1, and p85 overexpression by an IHC score 2+ and 3+.

### Statistical analysis

Relationships between tumor changes (expressed as mutational or expression status) and clinical, histological and biological parameters were estimated with the Chi^2^ test. A level of significance was set at 5%. Metastasis-free survival (MFS) was determined as the interval between diagnosis and detection of the first metastasis. Survival distributions were estimated by the Kaplan-Meier method [24], and the significance of differences between survival rates was ascertained with the log-rank test [25]. Cox’s proportional hazards regression model [26] was used to assess prognostic significance in multivariate analysis.

## Results

### *PIK3CA, PIK3R1* and *AKT1* mutational analysis

The present study extends our previously published data describing the positive effect of *PIK3CA* exon 9 and 20 mutations on breast cancer patient survival [12]. In the present study, *PIK3CA* mutations were additionally assessed in exons 1 and 2. *PIK3CA* mutations were identified in 151 (33.0%) of the 458 samples, in line with previous studies in which *PIK3CA* mutations were found in 10 to 40% of breast cancer cases [3,4,8]. Sixty-three tumors showed *PIK3CA* mutations located in exon 9, 85 tumors showed mutations in exon 20, and one tumor showed mutations in both exon 9 and exon 20. Five mutations were found in exon 1, including two cases with 3 nucleotide deletions (c.305_307del and c.328_330del). Three other mutated tumors showed point mutations (R115L in one case and R108H in two cases). Two tumors showed mutations in exon 2 (both G118D). Point mutations in exons 1 and 2 were always found in cases mutated in either exon 9 or exon 20, but the two tumors with deletions did not present any additional *PIK3CA* mutations in other exons. Breast cancer subgroup analysis demonstrated *PIK3CA* mutations with the lowest frequency (10/69; 14.5%) in HR-/ERBB2- tumors and the highest frequency (118/290; 40.7%) in HR+/ERBB2- tumors, while an intermediate frequency of *PIK3CA* mutations was observed in HR-/ERBB2+ and HR+/ERBB2+ tumors (9/45; 20.0% and 14/54; 25.9%, respectively).

*PIK3R1* mutations were screened in exons 11–15 and were present in 10 (2.2%) of the 454 available samples (Additional file 3: Table S3). Seven cases of deletions of 3-nucleotide multiples were observed in exons 11 and 13 (in the area between nucleotides 1345–1368 and 1701–1743, respectively), 2 cases of duplications of 3-nucleotide multiples were observed in exon 13 (in the area between nucleotides 1650–1723) and 1 case of point mutations were observed in exon 15 (c.1925G > T). It is noteworthy that we found also c.1590G > A giving the AAG --> AAA (Lys) nucleotide substitution located in exon 13 that is probably a polymorphism with no amino acid change. *PIK3R1* mutations were found in only 1 of the 151 *PIK3CA*-mutated cases and in 10 of the 297 *PIK3CA* wild-type cases. The low frequency of *PIK3R1* mutations did not allow any further statistical analysis concerning a possible association between *PIK3R1* mutations and clinical, histological and biological parameters.

*AKT1* mutation (E17K) was found in 15 (3.3%) of the 457 available samples. *AKT1* mutations were found in only 1 of the 161 *PIK3CA/PIK3R1*-mutated cases and 14 of the 297 *PIK3CA/PIK3R1* wild-type cases and tended therefore to mutual exclusivity with *PI3K* mutations (p = 0.019).

Altogether, we observed *PIK3CA* and/or *PIK3R1* and/or *AKT1* mutations in 174/454 (38.3%) breast cancer tumors. Breast cancer subgroup analysis demonstrated mutation of at least one of the three genes with the highest frequency in HR+/ERBB2- tumors (133/289; 46.0%). The other 3 breast cancer subtypes showed a lower frequency of these mutations: HR+/ERBB2+ in 15/54 (27.8%), HR-/ERBB2+ in 10/43 (23.3%) and HR-/ERBB2- in 16/68 (23.5%).

### mRNA expression

The *PIK3CA*, *PIK3R1* and *AKT1* mRNA expression levels were assessed in the whole series of 458 samples. *PIK3R1* underexpression was found in 283 (61.8%) cases, indicating a relevant tumor alteration occurring in the majority of tumor samples (Table 1). Moreover, when assessing breast cancer subgroups, *PIK3R1* was predominantly underexpressed in HR-/ERBB2- and HR-/ERBB2+ tumors (p < 0.0000001) (Table 2), while *PIK3CA* was deregulated in only a minority of tumor samples: overexpressed in 18 (3.9%) and underexpressed in 40 (8.7%) cases (Table 1). *PIK3CA* expression did not vary significantly between the four breast cancer subgroups based on hormone and ERBB2 receptor status (Table 2). Expression levels of *PIK3CA*, the oncogene bearing the highest number of mutations in breast cancer, were therefore mostly stable in breast cancer subgroups indicating that mutations constituted the main tumor change affecting *PIK3CA*. These results show that changes of expression of *PIK3R1* but not *PIK3CA* play a role in breast cancer, specifically in hormone receptor-negative cases.

**Table 1:** Gene mRNA levels in 458 breast tumors

**Genes**	**Median Ct of normal breast tissue (n = 10)**	**Normal breast tissue (n = 10)**	**Breast tumors n = 458**	**Percentage of underexpressed tumors (Ntarget ≤0.5)**	**Percentage of normal expressed tumors**	**Percentage of overexpressed tumors (Ntarget ≥2)**	**Percentage of overexpressed tumors (Ntarget ≥5)**
***EGFR***	30.2 (29.3-31.5)^a^	1.0 (0.7 -1.3)^b^	0.2 (0.0-112.9)^b^	**84.9%**^c^	**13.3%**^c^	1.8%^c^	0.7%^c^
***PIK3CA***	29.7 (28.4-31.0)	1.0 (0.7-1.3)	0.9 (0.2-33.4)	8.7%	**87.4%**	3.9%	0.7%
***PIK3R1***	26.8 (25.8-28.1)	1.0 (0.7-1.5)	0.4 (0.0-5.2)	**61.8%**	**36.0%**	2.2%	0.2%
***PDK1***	31.8 (29.7-33.5)	1.0 (0.5-1.9)	1.0 (0.0-14.7)	**13.3%**	**69.0%**	**17.7%**	2.2%
***PTEN***	26.4 (25.3-31.3)	1.1 (0.7-2.0)	0.8 (0.1-9.0)	**17.0%**	**81.0%**	2.0%	0.4%
***AKT1***	28.7 (27.5-30.1)	1.0 (0.7-1.5)	1.5 (0.0-11.1)	1.3%	**73.4%**	**25.3%**	2.8%
***AKT2***	26.7 (25.4-29.7)	1.0 (0.7-2.0)	1.7 (0.5-12.2)^d^	0.0%	**64.0%**	**36.0%**	3.3%
***AKT3***	26.0 (23.8-28.4)	1.0 (0.6-1.9)	0.4 (0.0-7.5)^d^	**67.1%**	**31.1%**	1.8%	0.2%
***GOLPH3***	27.9 (26.4-29.0)	1.0 (0.8-1.6)	1.4 (0.3-6.7)	0.7%	**79.9%**	**19.4%**	0.9%
***P70S6K***	31.2 (29.9-32.7)	1.0 (0.7-1.8)	1.2 (0.0-19.6)	2.2%	**79.7%**	**18.1%**	3.7%
***WEE1***	28.4 (26.1-29.8)	1.0 (0.5-1.6)	0.8 (0.2-6.9)	**18.3%**	**77.3%**	4.4%	0.2%

^a^Median (range) of gene Ct values.

^b^Median (range) of gene mRNA levels; the mRNA values of the samples were normalized so that the median of the 10 normal breast tissue mRNA values was 1.

^c^Percentages of underexpressing, normal and overexpressing tumors using cut-offs of N*target* ≤0.5 and N*target***≥**2.

^d^Data available in 456 samples.

The bold numbers stress important finding or overall characterisation of a group or p-value.

**Table 2:** Genes mRNA levels in the 4 breast tumor subtypes

	***All tumors***	***Tumor subtypes***				
		***HR-ERBB2-***	***HR-ERBB2+***	***HR + ERBB2-***	***HR + ERBB2+***	***P-value***^***a***^
	*n = 458*	*n = 69*	*n = 45*	*n = 290*	*n = 54*	
*PIK3CA values: median [range]*	*0.9 (0.2-33.4)*	*0.9 (0.3-33.4)*	*0.7 (0.3-1.7)*	*0.9 (0.2-5.9)*	*1.0 (0.4-5.6)*	*NS*
*Underexpressed tumors (%)*	**40 (8.7)**	*6 (8.7)*	*5 (11.1)*	*25 (8.6)*	*4 (7.4)*	
*Non-underexpressed tumors (%)*	418 (91.3)	*63 (91.3)*	*40 (88.9)*	*265 (91.4)*	*50 (92.6)*	
*PIK3R1 values: median [range]*	*0.4 (0.0-5.2)*	*0.2 (0.0-2.2)*	*0.3 (0.1-1.5)*	*0.5 (0.1-4.4)*	*0.4 (0.1-5.2)*	***<0.0000001***
*Underexpressed tumors (%)*	**283 (61.8)**	*61 (88.4)*	*40 (88.9)*	*150 (51.7)*	*32 (59.3)*	
*Non-underexpressed tumors (%)*	175 (38.2)	*8 (11.6)*	*5 (11.1)*	*140 (48.3)*	*22 (40.7)*	
*PDK1 values: median [range]*	*1.0 (0.0-14.7)*	*2.4 (0.5-14.7)*	*1.7 (0.4-6.2)*	*0.9 (0.0-3.3)*	*0.9 (0.1-2.4)*	***<0.0000001***
*Underexpressed tumors (%)*	**61 (13.3)**	*0*	*1 (2.2)*	*51 (7.9)*	*9 (16.6)*	
*Normally expressed tumors (%)*	316 (69.0)	*25 (36.2)*	*27 (60.0)*	*221 (90.0)*	*43 (79.6)*	
*Overexpressed tumors (%)*	**81 (17.7)**	*44 (63.8)*	*17 (37.8)*	*18 (2.1)*	*2 (3.7)*	
*PTEN values: median [range]*	*0.8 (0.1-9.0)*	*0.6 (0.1-1.5)*	*0.8 (0.3-1.9)*	*0.8 (0.1-9.0)*	*0.9 (0.4-3.3)*	***0.0000066***
*Underexpressed tumors (%)*	**78 (17.0)**	*27 (39.1)*	*6 (13.3)*	*39 (13.4)*	*6 (11.1)*	
*Non-underexpressed tumors (%)*	380 (83.0)	*42 (60.9)*	*39 (86.7)*	*251 (86.6)*	*48 (88.9)*	
*AKT1 values: median [range]*	*1.5 (0.0-11.1)*	*1.1 (0.0-11.1)*	*2.0 (0.6-10.0)*	*1.4 (0.4-6.1)*	*1.8 (0.6-9.9)*	***0.00019***
*Non-overexpressed tumors (%)*	342 (74.7)	*55 (79.7)*	*24 (53.3)*	*230 (79.3)*	*33 (61.1)*	
*Overexpressed tumors (%)*	**116 (25.3)**	*14 (20.3)*	*21 (46.7)*	*60 (20.7)*	*21 (38.9)*	
*AKT2 values: median [range]*^b^	*1.7 (0.5-12.2)*	*1.7 (0.7-12.2)*	*1.4 (0.8-8.7)*	*1.8 (0.5-10.6)*	*1.8 (0.5-7.0)*	***0.0097***
*Non-overexpressed tumors (%)*	293 (64.3)	*46 (67.6)*	*38 (84.4)*	*180 (62.3)*	*29 (53.7)*	
*Overexpressed tumors (%)*	**163 (35.7)**	*22 (32.4)*	*7 (15.6)*	*109 (37.7)*	*25 (46.3)*	
*AKT3 values: median [range]*^b^	*0.4 (0.0-7.5)*	*0.5 (0.0-2.2)*	*0.3 (0.1-0.9)*	*0.4 (0.0-7.5)*	*0.4 (0.1-2.3)*	*NS*
*Underexpressed tumors (%)*	**306 (67.1)**	*38 (55.9)*	*32 (71.1)*	*198 (68.5)*	*38 (70.4)*	
*Non-underexpressed tumors (%)*	150 (32.9)	*30 (44.1)*	*13 (28.9)*	*91 (31.5)*	*16 (29.6)*	
*GOLPH3 values: median [range]*	*1.4 (0.3-6.7)*	*1.2 (0.6-3.3)*	*1.4 (0.7-5.0)*	*1.3 (0.3-6.7)*	*1.7 (0.8-5.4)*	*NS*
*Non-overexpressed tumors (%)*	369 (80.6)	*54 (78.3)*	*36 (80.0)*	*241 (83.1)*	*38 (70.4)*	
*Overexpressed tumors (%)*	**89 (19.4)**	*15 (21.7)*	*9 (20.0)*	*49 (16.9)*	*16 (29.6)*	
*P70S6K values: median [range]*	*1.2 (0.0-19.6)*	*1.0 (0.0-5.4)*	*1.9 (0.6-9.9)*	*1.2 (0.3-8.0)*	*1.4 (0.4-19.6)*	***<0.0000001***
*Non-overexpressed tumors (%)*	375 (81.9)	*64 (92.8)*	*24 (53.3)*	*250 (86.2)*	*37 (68.5)*	
*Overexpressed tumors (%)*	**83 (18.1)**	*5 (7.2)*	*21 (46.7)*	*40 (13.8)*	*17 (31.5)*	
*WEE1 values: median [range]*	*0.8 (0.2-6.9)*	*0.7 (0.2-6.9)*	*0.9 (0.3-2.8)*	*0.8 (0.2-3.9)*	*0.8 (0.3-4.1)*	***0.0014***
*Underexpressed tumors (%)*	**84 (18.3)**	*24 (34.8)*	*6 (13.3)*	*43 (14.8)*	*11 (20.4)*	
*Non-underexpressed tumors (%)*	374 (81.7)	*45 (65.2)*	*39 (86.7)*	*247 (85.2)*	*43 (79.6)*	

^a^Chi^2^ test of independency in contingency tables (tumor subtype vs. gene expression). NS: not significant.

^b^Data available in 456 samples.

The bold numbers stress important finding or overall characterisation of a group or p-value.

*AKT1* overexpression was present in 116 (25.3%) of the 458 available samples, mostly in HR-/ERBB2+ and HR+/ERBB2+ tumors (p = 0.00019) (Table 2). Seven of the 15 *AKT1* mutated tumors also showed increased *AKT1* expression. However, *AKT1* mutation and expression status as well as expression changes in other genes of the PI3K/AKT pathway did not show any statistically significant association (data not shown) possibly because of the small number of *AKT1* mutated cases.

mRNA expression levels of other genes involved in the PI3K/AKT pathway were also evaluated., i.e. *EGFR*, *PDK1*, *PTEN*, *AKT2* and *3*, *GOLPH3*, *P70S6K*, and *WEE1* (Table 1). Markedly high expression that might be caused by gene amplification was observed only in low frequency (<4%) of tumors as shows the last colon in the Table 1. *PTEN* underexpression was significantly mutually exclusive with *PIK3CA*, *PIK3R1* and *AKT1* mutations (p = 0.00016), as it was observed in only one *AKT1* mutated tumor and 14 *PIK3CA* mutated tumors. Expression levels were also compared in the four breast cancer subgroups as shown in Table 2. Interestingly, gene expressions were deregulated in different ways in the 4 subgroups. *EGFR* underexpression was demonstrated in all subgroups, as previously published [27]. *P70S6K* and *AKT1* was predominantly overexpressed in ERBB2+ tumors (p < 0.0000001 and 0.00019, respectively). This increased expression of these two genes might be linked to the PI3K/AKT pathway activated by ERBB2 overexpression. On the other hand, expression changes in HR-/ERBB2- tumors might indicate downstream activation of the pathway occurring despite the negativity of ERBB2. The 4 molecular subgroups of breast cancer therefore appeared to undergo distinct changes at the levels of mRNA expression of the genes involved in the PI3K/AKT pathway. These data would benefit from confirmation at protein level (both quantity and activity).

The next step of analysis focused on PI3K constituents, specifically *PIK3R1* expression and *PIK3CA* mutations in relation to expression levels of the other genes evaluated. Tumors characterized by *PIK3R1* underexpression were associated with deregulation of other genes involved in the PI3K/AKT pathway (Table 3). *PIK3R1* underexpression was negatively associated with *PIK3CA* mutations (p = 0.00097) and these two parameters were therefore predominantly mutually exclusive. In contrast to *PIK3R1*, deregulation of the expression of genes involved in the PI3K/AKT pathway was almost exclusively associated with *PIK3CA* wild-type tumors (Table 4).

**Table 3:** **Comparison of *PIK3R1 *** **expression status and alterations of other genes of interest**

		**Number of patients (%)**		
	**Total population (%)**	***PIK3R1 *** **underexpression**	***PIK3R1 *** **non-underexpression**	***P-value***^***a***^
*Total*	*458 (100.0)*	*283 (61.8)*	*175 (38.2)*	
*EGFR values: median [range]*	*0.2 (0.0-112.9)*	*0.1 (0.0-7.3)*	*0.2 (0.0-112.9)*	
Underexpressed tumors (%)	**389 (84.9)**	250 (88.3)	139 (79.4)	***0.0096***
Non-underexpressed tumors (%)	69 (15.1)	33 (11.7)	36 (20.6)	
*PDK1 values: median [range]*	*1.0 (0.0-14.7)*	*1.2 (0.1-14.7)*	*0.9 (0.0-6.2)*	
Underexpressed tumors (%)	**61 (13.3)**	26 (9.2)	35 (20.0)	***0.000004***
Normally expressed tumors (%)	316 (69.0)	189 (66.8)	127 (72.6)	
Overexpressed tumors (%)	**81 (17.7)**	68 (24.0)	13 (7.4)	
*AKT1 values: median [range]*	*1.5 (0.0-11.1)*	*1.4 (0.4-10.0)*	*1.6 (0.0-11.1)*	
Non-overexpressed tumors (%)	342 (74.7)	216 (76.3)	126 (72.0)	*NS*
Overexpressed tumors (%)	**116 (25.3)**	67 (23.7)	49 (28.0)	
*AKT2 values: median [range]*^*b*^	*1.0 (0.7-2.3)*	*1.6 (0.5-10.6)*	*1.8 (0.5-12.2)*	
Non-overexpressed tumors (%)	293 (64.3)	189 (67.0)	104 (59.8)	*NS*
Overexpressed tumors (%)	**163 (35.7)**	93 (33.0)	70 (40.2)	
*AKT3 values: median [range]*^*b*^	*1.0 (0.4-1.9)*	*0.3 (0.0-2.4)*	*0.5 (0.1-7.5)*	
Underexpressed tumors (%)	**306 (67.1)**	215 (76.2)	91 (52.3)	***0.00000013***
Non-underexpressed tumors (%)	150 (32.9)	67 (23.8)	83 (47.7)	
*GOLPH3 values: median [range]*	*1.4 (0.3-6.7)*	*1.3 (0.3-5.2)*	*1.7 (0.7-6.7)*	
Non-overexpressed tumors (%)	369 (80.6)	242 (85.5)	127 (72.6)	***0.00067***
Overexpressed tumors (%)	**89 (19.4)**	41 (14.5)	48 (27.4)	
*P70S6K values: median [range]*	*1.2 (0.0-19.6)*	*1.2 (0.4-19.6)*	*1.2 (0.0-8.9)*	
Non-overexpressed tumors (%)	375 (81.9)	226 (79.9)	149 (85.1)	*NS*
Overexpressed tumors (%)	**83 (18.1)**	57 (20.1)	26 (14.9)	
*WEE1 values: median [range]*	*0.8 (0.2-6.9)*	*0.7 (0.2-4.1)*	*0.9 (0.2-6.9)*	
Underexpressed tumors (%)	**84 (18.3)**	68 (24.0)	16 (9.1)	***0.000063***
Non-underexpressed tumors (%)	374 (81.7)	215 (76.0)	159 (90.9)	
*PTEN values: median [range]*	*0.8 (0.1-9.0)*	*0.7 (0.1-9.0)*	*1.0 (0.4-5.8)*	
Underexpressed tumors (%)	**78 (17.0)**	71 (25.1)	7 (4.0)	***< 0.0000001***
Non-underexpressed tumors (%)	380 (83.0)	212 (74.9)	168 (96.0)	
*PIK3CA*				
Wild-type (%)	307 (67.0)	*205 (72.4)*	*102 (58.3)*	***0.0017***
Mutation (%)	**151 (33.0)**	*78 (27.6)*	*73 (41.7)*	
*PIK3R1*^*c*^				
Wild-type (%)	444 (97.8)	*276 (98.6)*	*168 (96.6)*	*NS*
Mutation (%)	**10 (2.2)**	*4 (1.4)*	*6 (3.4)*	
*AKT1*^*d*^				
Wild-type (%)	442 (96.7)	272 (96.5)	170 (97.1)	*NS*
Mutation (%)	**15 (3.3)**	10 (3.5)	5 (2.9)	

^a^Chi^2^ test. NS: not significant.

^b^Data available in 456 samples.

^c^Data available in 454 samples.

^d^Data available in 457 samples.

The bold numbers stress important finding or overall characterisation of a group or p-value.

**Table 4:** **Comparison of *PIK3CA *** **mutational status and alterations in other genes of interest**

		**Number of patients (%)**		
	**Total population (%)**	***PIK3CA *** **wild-type**	***PIK3CA-*** **mutated**	***P-value***^***a***^
*Total*	*458 (100.0)*	*307 (67.0)*	*151 (33.0)*	
*EGFR values: median [range]*	*0.2 (0.0-112.9)*	*0.2 (0.0-112.9)*	*0.2 (0.0-7.3)*	
Underexpressed tumors (%)	**389 (84.9)**	256 (83.4)	133 (88.1)	*NS*
Non-underexpressed tumors (%)	69 (15.1)	51 (16.6)	18 (11.9)	
*PIK3R1 values: median [range]*	*0.4 (0.0-5.2)*	*0.3 (0.0-4.4)*	*0.5 (0.1-5.2)*	
Underexpressed tumors (%)	**283 (61.8)**	205 (66.8)	78 (51.7)	***0.0017***
Non-underexpressed tumors (%)	175 (38.2)	102 (33.2)	73 (48.3)	
*PDK1 values: median [range]*	*1.0 (0.0-14.7)*	*1.1 (0.0-14.7)*	*0.8 (0.1-4.5)*	
Underexpressed tumors (%)	**61 (13.3)**	35 (11.4)	26 (17.2)	***0.0011***
Normally expressed tumors (%)	316 (69.0)	204 (66.5)	112 (74.2)	
Overexpressed tumors (%)	**81 (17.7)**	68 (22.1)	13 (8.6)	
*PTEN values: median [range]*	*0.8 (0.1-9.0)*	*0.8 (0.1-5.8)*	*0.9 (0.1-9.0)*	
Underexpressed tumors (%)	**78 (17.0)**	64 (20.8)	14 (9.3)	***0.0019***
Non-underexpressed tumors (%)	380 (83.0)	243 (79.2)	137 (90.7)	
*AKT1 values: median [range]*	*1.5 (0.0-11.1)*	*1.5 (0.0-11.1)*	*1.5 (0.4-9.9)*	
Non-overexpressed tumors (%)	342 (74.7)	230 (74.9)	112 (74.2)	*NS*
Overexpressed tumors (%)	**116 (25.3)**	77 (25.1)	39 (25.8)	
*AKT2 values: median [range]*^*b*^	*1.0 (0.7-2.3)*	*1.7 (0.5-12.2)*	*1.6 (0.5-10.6)*	
Non-overexpressed tumors (%)	293 (64.3)	190 (62.3)	103 (68.2)	*NS*
Overexpressed tumors (%)	**163 (35.7)**	115 (37.7)	48 (31.8)	
*AKT3 values: median [range]*^*b*^	*1.0 (0.4-1.9)*	0.4 (0.0-3.6)	*0.4 (0.1-7.5)*	
Underexpressed tumors (%)	**306 (67.1)**	206 (67.5)	100 (66.2)	*NS*
Non-underexpressed tumors (%)	150 (32.9)	99 (32.5)	51 (33.8)	
*GOLPH3 values: median [range]*	*1.4 (0.3-6.7)*	*1.4 (0.5-6.7)*	*1.3 (0.3-5.4)*	
Non-overexpressed tumors (%)	369 (80.6)	242 (78.8)	127 (84.1)	*NS*
Overexpressed tumors (%)	**89 (19.4)**	65 (21.2)	24 (15.9)	
*P70S6K values: median [range]*	*1.2 (0.0-19.6)*	*1.2 (0.0-19.6)*	*1.1 (0.4-8.0)*	
Non-overexpressed tumors (%)	375 (81.9)	233 (75.9)	142 (94.0)	***0.0000022***
Overexpressed tumors (%)	**83 (18.1)**	74 (24.1)	9 (6.0)	
*WEE1 values: median [range]*	*0.8 (0.2-6.9)*	*0.8 (0.2-6.9)*	*0.7 (0.3-3.4)*	
Underexpressed tumors (%)	**84 (18.3)**	61 (19.9)	23 (15.2)	*NS*
Non-underexpressed tumors (%)	374 (81.7)	246 (80.1)	128 (84.8)	
*PIK3R1*^*c*^				
Wild-type (%)	444 (97.8)	294 (97.0)	150 (99.3)	*NS*
Mutation (%)	**10 (2.2)**	9 (3.0)	1 (0.7)	
*AKT1*^*d*^				
Wild-type (%)	442 (96.7)	292 (95.4)	150 (99.3)	*NS*
Mutation (%)	**15 (3.3)**	14 (4.6)	1 (0.7)	

^a^Chi^2^ test. NS: not significant.

^b^Data available in 456 samples.

^c^Data available in 454 samples.

^d^Data available in 457 samples.

The bold numbers stress important finding or overall characterisation of a group or p-value.

### Immunohistochemistry

**Figure 1:**
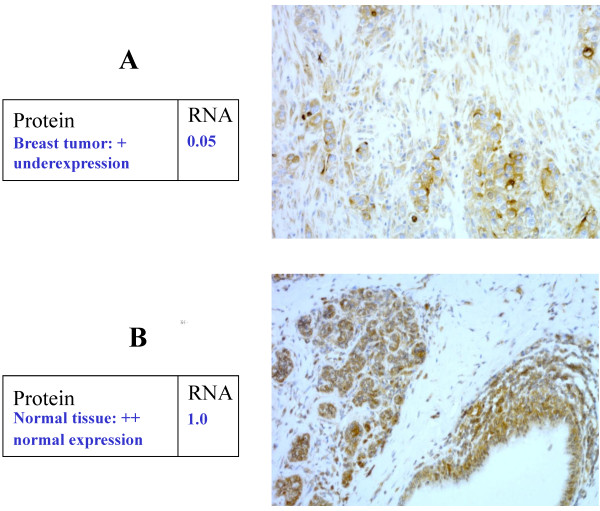
**Comparison of *PIK3R1*/p85 immunohistochemistry and mRNA expression results. A**. Tumor sample with protein underexpression (expression intensity +) and decreased mRNA expression (normalized mRNA expression value 0.05). **B**. Healthy tissue sample with normal protein expression (expression intensity +++) and normal mRNA expression (normalized mRNA expression value 1.0).

Alteration of p85 (encoded by *PIK3R1*) and PTEN expression was also verified at the protein level by immunohistochemistry in randomly selected samples with low and high mRNA expression. In both cases, samples showing decreased mRNA expression (5 *PIK3R1* underexpressed- and 5 *PTEN* underexpressed-tumors) also presented low immunohistochemical staining intensity. Similarly, samples showing normal mRNA expression (7 *PIK3R1* expressing and 8 *PTEN* expressing tumors) presented strong immunohistochemical staining intensity. The only exceptions were two samples stained for PTEN (one showing low mRNA expression and more intense immunohistochemistry staining, the other showing opposite features). A good match (23/25 samples tested) was therefore obtained between mRNA and protein expression status for both *PIK3R1* and *PTEN* (Figure 1). These results suggest that the regulation of p85 (and PTEN) expression is mainly transcriptional.

### Survival analysis

**Figure 2:**
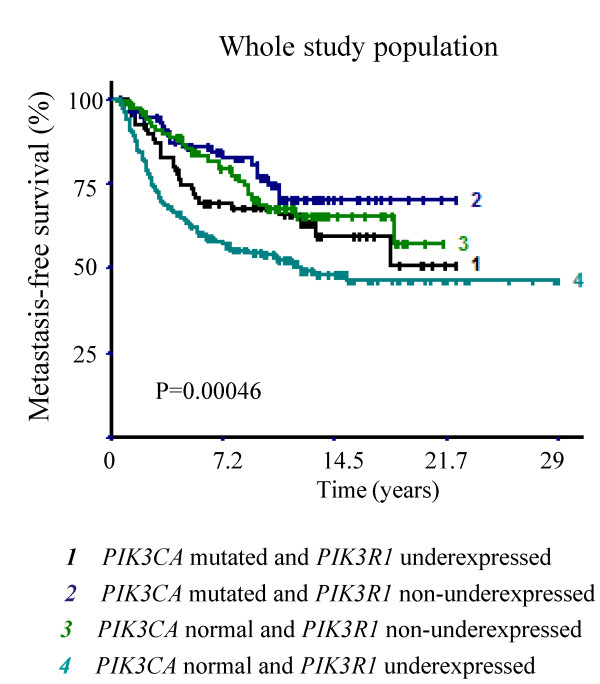
**Survival curves of four patient groups according to *PIK3R1 *** **expression status and *PIK3CA *** **mutations.**

Survival curves were compared to assess the possible impact of these expression changes and mutations on patient outcome. Additional file 4: Table S4 summarizes survival analysis performed on the overall patient series. Patients presenting any of the mutations assessed in this study (*PIK3CA*, *PIK3R1* or *AKT1*) had a significantly better MFS (p = 0.024). Among the 11 genes studied, only *PIK3CA* mutations and *PIK3R1* underexpression, as separate markers, were associated with MFS and had opposite effects on patient survival: *PIK3CA* mutation was associated with better MFS and *PIK3R1* underexpression was associated with poorer MFS (p = 0.016 and p = 0.00028, respectively). *PIK3R1* underexpression was associated with histological grade 3 status and an increased rate of positive axillary lymph nodes (p < 0.0000001 and p = 0.013, respectively). HR- and ERBB2+ tumors were also more likely to present *PIK3R1* underexpression (p < 0.0000001 and p = 0.011, respectively). These results show that *PIK3R1* underexpression predominantly occurred in tumors with poorer prognostic markers (Additional file 5: Table S5). The combination of these two molecular markers (*PIK3CA* mutations and *PIK3R1* underexpression) can be considered to provide more accurate prediction of patient survival than when they are considered separately. Combined analysis of *PIK3CA* mutations and *PIK3R1* expression status defined four separate prognostic groups with significantly different survivals. Comparison of all four survival curves showed statistical differences with p = 0.00046 (log-rank test for 4-level factor, Figure 2). The least favorable survival was observed in the subgroup characterized by *PIK3CA* wild-type and *PIK3R1* underexpression and the most favorable survival was observed in the subgroup characterized by *PIK3CA* mutation without *PIK3R1* underexpression.

Multivariate analysis using a Cox proportional hazards model (Table 5) assessed the predictive value for MFS of the parameters found to be significant on univariate analysis (i.e., Scarff-Bloom-Richardson histological grade, lymph node status, macroscopic tumor size, and ERα, PR, and ERBB2 status, as well as *PIK3CA* mutation and *PIK3R1* expression status). This analysis confirmed a trend towards an independent prognostic significance of *PIK3CA* mutations only in ERBB2+ tumors (p = 0.051). Furthermore, the prognostic significance of *PIK3R1* underexpression persisted in the overall series (p = 0.0013) and in breast cancer subgroups characterized by ERα + (p = 0.0076), PR + (p = 0.043), ERBB2+ (p = 0.018) and also ERBB2- (p = 0.024).

**Table 5:** Results of Cox multivariate analysis

**Variables**	**Total population**		**ER-positive patients**		**ER-negative patients**		**PR-positive patients**		**PR-negative patients**		**ERBB2-positive patients**		**ERBB2-negative patients**	
	**HR (95% CI)**^**a**^	***P*-value**^**b**^	**HR (95% CI)**^**a**^	***P*-value**^**b**^	**HR (95% CI)**^**a**^	***P*-value**^**b**^	**HR (95% CI)**^**a**^	***P*-value**^**b**^	**HR (95% CI)**^**a**^	***P*-value**^**b**^	**HR (95% CI)**^**a**^	***P*-value**^**b**^	**HR (95% CI)**^**a**^	***P*-value**^**b**^
*SBR*														
I	1	**0.041**	1	**0.018**		NS	1	**0.00068**		NS		NS	1	**0.0034**
II	1.27 (1.01-1.61)		1.42 (1.06-1.90)				1.87 (1.30-2.68)						1.49 (1.14-1.95)	
III	1.62 (1.02-2.59)		2.02 (1.13-3.61)				3.49 (1.70-7.17)						2.23 (1.31-3.82)	
*pN*														
0	1	**0.00021**	1	**0.0024**	1	**0.02**	1	**0.0097**	1	**0.0055**	1	**0.0000076**		NS
1-3	1.54 (1.22-1.93)		1.53 (1.16-2.01)		1.61 (1.08-2.40)		1.57 (1.12-2.22)		1.53 (1.13-2.07)		2.91 (1.82-4.64)			
>3	2.36 (1.50-3.72)		2.34 (1.35-4.04)		2.58 (1.16-5.74)		2.47 (1.24-4.91)		2.34 (1.28-4.28)		8.47 (3.32-21.57)			
*pT*														
≤25 mm	1	**0.004**	1	**0.00014**		NS	1	**0.00096**		NS		NS	1	**0.00034**
>25 mm	1.62 (1.17-2.24)		2.16 (1.45-3.20)				2.22 (1.38-3.55)						1.97 (1.36-2.86)	
*ER*				-		-								
*Negative*		NS						NS		NS		NS		NS
*Positive*														
*PR*								-		-				
*Negative*		NS		NS		NS						NS		NS
*Positive*														
*ERBB2*												-		-
*Negative*		NS		NS		NS		NS		NS				
*Positive*														
*PIK3R1*														
*Underexpression*	1	**0.0013**	1	**0.0076**		NS	1	**0.043**		NS	1	**0.018**	1	**0.024**
*Normal*	0.59 (0.43-0.81)		0.62 (0.44-0.88)				0.65 (0.43-0.99)				0.40 (0.19-0.85)		0.66 (0.46-0.95)	
*Overexpression*	0.35 (0.18-0.66)		0.38 (0.19-0.77)				0.42 (0.18-0.97)				0.16 (0.03-0.73)		0.44 (0.21-0.89)	
*PI3KCA*														
*Normal*		NS		NS		NS		NS		NS	1	**0.051**		NS
*Mutated*											0.39 (0.15-1.00)			

^a^Hazard ratio (95% Confidential Interval). NS: not significant.

^b^Multivariate Cox analysis. NS: not significant.

The bold numbers stress important finding or overall characterisation of a group or p-value.

## Discussion

This study extends the previously obtained data concerning the positive prognostic role of exon 9 and 20 *PIK3CA* mutations in breast cancer [12]. This study focused on PI3K signaling pathway, particularly the two subunits of PI3K encoded by *PIK3CA* and *PIK3R1* genes. In addition to our previous study, *PIK3CA* mutations were also assessed in exons 1 and 2 that have been recently shown to be frequently mutated in endometrial cancer [15]. *PIK3CA* mutations were detected in 33.0% of cases (exons 1, 2, 9, 20) and *PIK3R1* mutations were detected in 2.2% of cases (exons 11, 12, 13, 15). The low frequency of about 3% *PIK3R1* mutations is in agreement with published studies [17,28]. *AKT1* mutations (exon 4) were also assessed and detected in 3.3% of tumors. This finding is also in agreement with previous studies describing a moderate frequency of *AKT1* mutations in breast cancer and their association with positive hormone receptor status [6]. *PIK3CA*, *PIK3R1* and *AKT1* mutations were mutually exclusive and were observed in a total of 175 breast cancer tumors. Interestingly, *PIK3R1* underexpression was observed in 61.8% of breast cancer tumors. *PIK3CA* mutations were associated with better MFS and *PIK3R1* underexpression was associated with poorer MFS (p = 0.014 and p = 0.00028, respectively). By combining *PIK3CA* mutation and *PIK3R1* expression states, we identified four prognostic groups with significantly different MFS (p = 0.00046). These new results suggest that *PIK3CA* mutations and *PIK3R1* underexpression are associated with opposite prognostic impacts on breast cancer patient survival. Multivariate analysis showed that *PIK3R1* expression status was an independent predictor of MFS in the total population (p = 0.0013), whereas *PIK3CA* mutation status only showed a trend in the ERBB2+ population (p = 0.051).

The frequency and associations of genomic and protein expression alterations in the PI3K pathway differ in the various breast cancer subgroups. Additionally, some alterations may co-exist, while others are mutually exclusive. Mutually exclusive mutations have been previously reported for *PIK3CA* and *AKT1* mutations [4]. We and other teams have found *PIK3CA* mutations in 10 to 40% of breast cancer cases and *AKT1* mutations in less than 10% of cases [3-6,8,17]. Our data are in agreement with the mutational frequencies described by other authors. Our findings also support the data recently published by Ellis et al., who described a low frequency of exon 1 and 2 mutations in breast cancer. They also observed missense mutations in these two exons occurring in cases bearing additional *PIK3CA* mutations, whereas one deletion in exon 1 was not accompanied by another *PIK3CA* mutation [29]. The most frequent mutations were E542K and E545K in exon 9 and H1047R in exon 20 in keeping with most other studies [4,8,17,18]. We also found that *PIK3R1* mutations tended to mutual exclusivity with *PIK3CA* and *AKT1* mutations. PTEN loss occurring in up to 30% of unselected breast tumor cohorts is also predominantly mutually exclusive with *PIK3CA* and *AKT1* mutations [4,18]. *PIK3R1* mutations as well as combined mutations of the three genes studied were also found to be mutually exclusive with *PTEN* underexpression (p = 0.00016). As *PIK3CA* and *AKT1* are oncogenes activated by mutations and as *PIK3R1* and *PTEN* are tumor suppressors mainly inactivated by underexpression, respectively, all these alterations result in PI3K pathway activation. The frequencies of *PIK3CA*, *PIK3R1* and *AKT1* alteration differ according to breast cancer subtypes. *PIK3CA* mutations have been previously described to occur most frequently in HR + breast tumors [4,12]. The highest mutational frequency for all of the genes assessed in this study (*PIK3CA* and/or *PIK3R1* and/or *AKT1*) was observed in HR+/ERBB2- tumors (133/289; 46.0%), while mutations were observed in up to 28% of cases in other breast cancer subtypes. In terms of expression, *PIK3R1* was underexpressed in about 90% of HR- tumors, but only in about 55% of HR + breast cancers. Similarly, *PTEN* underexpression was observed in 40% of triple-negative tumors versus 13% in other breast cancer subtypes, suggesting different mechanisms underlining PI3K pathway deregulation in specific breast tumor subtypes.

The protein p85α encoded by the *PIK3R1* gene has been described to play an important role in PI3K pathway signaling by stabilizing the other PI3K subunit – p110α – encoded by *PIK3CA* gene [7,16,30]. Loss of the p85α tumor suppressor effect leads to downstream PI3K pathway activation. The impact of *PIK3R1* deregulation on pathway signaling could be caused by the impaired ability of interaction of the two subunits and loss of the inhibitory effect of p85α on p110α and PI3K activity [7,28]. *PIK3R1* has been reported to play a tumor suppressor role in hepatocellular cancer and this tumor suppressor effect is lost in the case of gene underexpression [11,16]. Mostly point mutations and deletions have been reported for *PIK3R1*, but much less frequently in breast cancer (<5% of cases) than in other cancer types, such as endometrial cancer (about 20% of cases) [15,28]. *PIK3R1* mutations were observed in 2.2% of cases in the present study. *PIK3R1* mutations and p85 loss have also been associated with PI3K pathway activation and increased oncogenic potential. However, the fact that *PIK3R1* mutations are rare in breast cancer indicates that *PIK3R1* mRNA/p85α expression loss is the main deregulation occurring in breast tumors, particularly in HR- breast tumors. Another player affecting the PI3K pathway activation is PTEN, a tumor suppressor phosphatase which negatively regulates the PI3K pathway. Loss of PTEN expression is frequently observed in various cancer types and in up to 30% of breast cancers, leading to PI3K pathway activation [4]. Interestingly, p85 has also been suggested to have a positive regulatory effect on PTEN function via stabilization of this protein [15,16]. *PTEN* underexpression was found in 17% cases in our series (39% in triple-negative tumors) and was associated with *PIK3CA* wild-type status and *PIK3R1* underexpression, in line with previous findings.

There is growing evidence in the literature concerning the favorable outcome of *PIK3CA*-mutated breast cancer, as supported by the results of this study [9-12]. These mutations are known to play an activating role in cell lines and animal models [13,14]. Several hypotheses are currently proposed to explain the favorable prognostic impact of *PIK3CA* mutations: 1, *PIK3CA* mutations, when they are the only hit to the PI3K signaling pathway, have a limited oncogenic potential; 2, *PIK3CA* mutations result in oncogene-induced senescence; 3, *PIK3CA* mutation-bearing cells are more sensitive to chemotherapy and/or other treatment modalities; 4, *PIK3CA* mutation-induced signaling triggers a negative feedback loop inhibiting lower levels of the pathway [8,31]. *PIK3CA* mutations might affect the PI3K/AKT pathway in different ways in patient tumors and cell lines. The difference between *PIK3CA* mutation-related activation of the pathway in cell lines or animal models and patient outcome could be related to the treatment received by patients, as suggested above. In contrast with the *PIK3CA* mutation-associated survival advantage in anti-ERBB2 untreated patients, *PIK3CA* mutations appear to predict resistance to treatment including ERBB2 inhibitors such as trastuzumab [32,33].

The present study demonstrates that *PIK3R1* underexpression is associated with decreased patient survival. Immunohistochemical analysis showed that *PIK3R1* transcripts are translated into p85 protein in epithelial tumor cells (Figure 1). A strong correlation was also demonstrated between *PIK3R1* mRNA underexpression and decreased p85 protein levels. Immunohistochemistry could be the method of choice to routinely determine p85 expression status. *PIK3R1* underexpressing tumors were also prone to accumulate other changes of the PI3K/AKT pathway, i.e. *PDK1* overexpression and *EGFR, AKT3, PTEN* and *WEE1* underexpressions. *PIK3R1* underexpression is therefore associated with additional pathway deregulation and possibly also with increased signaling activation. In a murine model with liver-specific *PIK3R1* loss, this condition led to development of aggressive hepatocellular cancer [16]. Loss of *PIK3R1* mRNA expression in cell lines was associated with a more migratory and more invasive phenotype of MCF-7-14 cells compared to the parental MCF-7 cell line [34]. Lu *et al.* described a gene expression signature including *PIK3R1* distinguishing between low- and high-risk stage I lung cancer. The authors found low *PIK3R1* expression in high-risk compared to low-risk lung cancers [35]. Studies concerning glioblastomas have also suggested that these tumors might be negatively influenced by *PIK3R1* expression at the level of cell lines and in terms of patient survival [36,37]. The recently observed role of *PIK3R1* expression deregulation in breast cancer survival needs to be further assessed, preferably in a prospective clinical study.

Our results suggest that *PIK3R1* could potentially become a clinically useful independent prognostic marker in breast cancer. *PIK3R1* underexpression (as well *PIK3CA* mutation) might also predict a favorable response to treatment with PI3K inhibitors or inhibitors of lower levels of the signaling pathway, such as mTOR inhibitors [14,28,38,39]. Finally, *PIK3R1* underexpression (and *PIK3CA* mutation) could be explored as predictors of resistance to treatment with ERBB2 inhibitors such as trastuzumab [12].

## Conclusions

*PIK3CA* and *PIK3R1* are genes encoding two subunits of the PI3K enzyme, p110α and p85α, respectively. The present study showed that alterations in these two genes have a complementary impact on breast cancer patient survival. There is growing evidence supporting *PIK3CA* mutations as good prognostic markers in breast cancer, but the negative impact of *PIK3R1* underexpression on patient survival has been less extensively studied. These two potential tumor markers warrant further assessment, preferably in prospective clinical studies.

## Abbreviations

PIK3CA: Phosphatidylinositol 3-kinase, catalytic, alpha polypeptide gene; PIK3R1: Phosphatidylinositol 3-kinase, regulatory subunit 1 (alpha); PI3K: Phosphatidylinositol 3-kinase; RFS: Relapse-free survival; ERα: Estrogen receptor alpha; PR: Progesterone receptor; HR: Hormone receptors; RT-PCR: Reverse transcriptase-polymerase chain reaction.

## Competing interests

The authors declare that they have no competing interests.

## Author’s contributions

IB conceived and designed the study, supervised the study and participated on data analysis and interpretation. SV, AS, DM and MT participated on data acquisition. MC participated on data analysis and interpretation and on writing and revising the manuscript. DM, CC, ER and FS participated on analysis and interpretation of data. RL participated on data analysis and interpretation and on writing and revising the manuscript. All authors read and approved the final maunscript.

## Pre-publication history

The pre-publication history for this paper can be accessed here:

http://www.biomedcentral.com/1471-2407/13/545/prepub

## Supplementary Material

Additional file 1: Table S1 Characteristics of the 458 primary breast tumors. Click here for file

Additional file 2: Table S2 Oligonucleotide primer sequences for RT-PCR analysis. Click here for file

Additional file 3: Table S3 List of *PIK3R1* mutations found in the present study. Click here for file

Additional file 4: Table S4 Relationship between gene status and MFS. Click here for file

Additional file 5: Table S5 Characteristics of the 458 primary breast tumors correlated with *PIK3R1* expression status. Click here for file
