# Supplementary material for: PIK3R1 underexpression is an independent prognostic marker in breast cancer
Source: BMC Cancer. 2013 Nov 14;13:545. doi: 10.1186/1471-2407-13-545 (PMC4225603; doi:10.1186/1471-2407-13-545)
Supplement: Additional file 1: Table S1 — Characteristics of the 458 primary breast tumors. [file 1471-2407-13-545-S1.pdf]

Additional Table 1. Characteristics of the 458 primary breast tumors.

	Number of patients (%)	Number of distant relapses (%)	*P-value*[a]
Total	*458 (100.0)*	*170 (37.1)*	
Age			
≤50	*99 (21.6)*	*38 (38.4)*	*NS*
>50	*359 (78.4)*	*132 (36.8)*	
SBR histological grade[b,c]			
I	*58 (12.6)*	*8 (13.8)*	
II	*230 (50.2)*	*84 (36.5)*	***0.000079***
III	*161 (35.2)*	*74 (46.0)*	
Lymph node status[d]			
0	*120 (26.2)*	*35 (29.2)*	
1-3	*237 (51.7)*	*77 (32.5)*	***0.00000064***
>3	*100 (21.8)*	*58 (58.0)*	
Macroscopic tumor size[e]			
≤25mm	*223 (48.7)*	*63 (28.3)*	***0.00002***
>25mm	*227 (49.6)*	*106 (46.7)*	
ERα status			
Negative	*119 (26.0)*	*51 (42.9)*	***0.0086***
Positive	*339 (74.0)*	*119 (35.1)*	
PR status			
Negative	*195 (42.6)*	*86 (44.1)*	***0.0011***
Positive	*263 (57.4)*	*84 (31.9)*	
ERBB2 status			
Negative	*359 (78.4)*	*128 (35.7)*	*NS*
Positive	*99 (21.6)*	*42 (42.4)*	
Molecular subtypes			
HR- ERBB2-	*69 (15.1)*	*27 (39.1)*	
HR- ERBB2+	*45 (9.8)*	*23 (51.1)*	***0.0087***
HR+ ERBB2-	*290 (63.3)*	*101 (34.8)*	
HR+ ERBB2+	*54 (11.8)*	*19 (35.2)*	

[a]Log-rank test. NS: not significant.
[b]Scarff-Bloom-Richardson classification.
[c]Information available for 449 patients.
[d]Information available for 457 patients.
[e]Information available for 450 patients.
