# Supplementary material for: PIK3R1 underexpression is an independent prognostic marker in breast cancer
Source: BMC Cancer. 2013 Nov 14;13:545. doi: 10.1186/1471-2407-13-545 (PMC4225603; doi:10.1186/1471-2407-13-545)
Supplement: Additional file 2: Table S2 — Oligonucleotide primer sequences for RT-PCR analysis. [file 1471-2407-13-545-S2.pdf]

Additional Table 2. Oligonucleotide primer sequences for RT-PCR analysis.

Gene	Oligonucleotide	Sequence	PCR Product Size (bp)[a]
EGFR	Upper primer<br>Lower primer	5' – GGAGAACTGCCAGAAACTGACC – 3'<br>5' – GCCTGCAGCACACTGGTTG – 3'	106
PIK3CA	Upper primer<br>Lower primer	5' – CCTGATCTTCCTCGTGCTGCTC – 3'<br>5' – ATGCCAATGGACAGTGTTCCTCTT – 3'	91
PIK3R1	Upper primer<br>Lower primer	5' – GATTCTCAGCAGCCAGCTCTGAT – 3'<br>5' – GCAGGCTGTCGTTCATTCCAT – 3'	91
PDK1	Upper primer<br>Lower primer	5' – TCCAGATAATCTTCTCAGGACACCAT – 3'<br>5' – CATAAATAGCTTTAGCATCCTCAGCA – 3'	119
PTEN	Upper primer<br>Lower primer	5' – GTGGCGGAACTTGCAATCCT – 3'<br>5' – ATGAACTTGTCTTCCCGTCGTGT – 3'	97
AKT1	Upper primer<br>Lower primer	5' – CCCAGGTCACGTCGGAGACT – 3'<br>5' – ACTCCATGCTGTCATCTTGGTCA – 3'	99
AKT2	Upper primer<br>Lower primer	5' – ACGGCTCCTTCATTGGGTACA – 3'<br>5' – CTTCATCAGCTGGCATTCTGCTA – 3'	98
AKT3	Upper primer<br>Lower primer	5' – AACAGAACGACCAAAGCCAAACACAT – 3'<br>5' – GCTTCTGTCCATTCTTCCCTTTCCTC – 3'	114
GOLPH3	Upper primer<br>Lower primer	5' – CCTCCAGAAACGGTCCAGAACT – 3'<br>5' – TTAATGGATTCCATGTCTCACCACTA – 3'	61
P70S6K	Upper primer<br>Lower primer	5' – AGGACGCGGGCTCTGAGGAT – 3'<br>5'– ATTTCTCACAATGTTCCATGCCAAGT – 3'	108
WEE1	Upper primer<br>Lower primer	5' – TACTCCGGATTCTTTGTTGCTTCAT – 3'<br>5' – GTCTTCACCACAGGAATCATTCCA – 3'	85

[a] Length of polymerase chain reaction products (base pairs).
