# Supplementary material for: PIK3R1 underexpression is an independent prognostic marker in breast cancer
Source: BMC Cancer. 2013 Nov 14;13:545. doi: 10.1186/1471-2407-13-545 (PMC4225603; doi:10.1186/1471-2407-13-545)
Supplement: Additional file 3: Table S3 — List of PIK3R1 mutations found in the present study. [file 1471-2407-13-545-S3.pdf]

Additional Table 3. List of *PIK3R1* mutations found in the present study.

Sample	*PIK3R1* mutation			*PIK3CA* mutation
	Nucleotide	Codon	Exon	
1	c.1345_1347del	p.Leu449del	11	no
2	c.1351_1368del	p.Glu451_Phe456del	11	no
3	c.1650_1688dup	p.Lys551_Met563dup	13	no
4	c.1701_1727del	p.Pro568_Thr576del	13	no
5	c.1718_1723dup	p.Arg574_Lys575insMetArg	13	p.His1047Arg
6	c.1723_1731del	p.Lys575_Arg577del	13	no
7	c.1727_1729del	p.Thr576del	13	no
8	c.1738_1743del	p.Tyr580_Leu581del	13	no
9	c.1738_1743del	p.Tyr580_Leu581del	13	no
10	c.1925G>T	p.Arg642Leu	15	no
