# Supplementary material for: PIK3R1 underexpression is an independent prognostic marker in breast cancer
Source: BMC Cancer. 2013 Nov 14;13:545. doi: 10.1186/1471-2407-13-545 (PMC4225603; doi:10.1186/1471-2407-13-545)
Supplement: Additional file 4: Table S4 — Relationship between gene status and MFS. [file 1471-2407-13-545-S4.pdf]

Additional Table 4. Relationship between gene status and MFS.

	Total population (%)	Number of relapses (%)	*P-value*[a]
Total	*458 (100.0)*	*170 (37.1)*	
EGFR			
Underexpression	389 (84.9)	*153 (39.3)*	*NS*
Non-underexpression	69 (15.1)	*17 (24.6)*	
PIK3R1			
Underexpression	283 (61.8)	*122 (43.1)*	***0.00028***
Non-underexpression	175 (38.2)	*48 (27.4)*	
PDK1			
Underexpression	61 (13.3)	*22 (36.1)*	
Normal expression	316 (69.0)	*116 (36.7)*	*NS*
Overexpression	81 (17.7)	*32 (39.5)*	
PTEN			
Underexpression	78 (17.0)	*31 (39.7)*	*NS*
Non-underexpression	380 (83.0)	*139 (36.6)*	
AKT1			
Non-overexpression	342 (74.7)	*123 (36.0)*	*NS*
Overexpression	116 (25.3)	*47 (40.5)*	
AKT2[b]			
Non-overexpression	293 (64.3)	*119 (40.6)*	*NS*
Overexpression	163 (35.7)	*50 (30.7)*	
AKT3[b]			
Underexpression	306 (67.1)	*120 (39.2)*	*NS*
Non-underexpression	150 (32.9)	*49 (32.7)*	
GOLPH3			
Non-overexpression	369 (80.6)	*131 (35.5)*	*NS*
Overexpression	89 (19.4)	*39 (43.8)*	
P70S6K			
Non-overexpression	375 (81.9)	*134 (35.7)*	*NS*
Overexpression	83 (18.1)	*36 (43.4)*	
WEE1			
Underexpression	84 (18.3)	*34 (40.5)*	*NS*
Non-underexpression	374 (81.7)	*136 (36.4)*	
PIK3CA			
Non-mutated (%)	307 (67.0)	*124 (40.4)*	***0.016***
Mutated (%)	151 (33.0)	*46 (30.5)*	
PIK3R1[c]			

Non-mutated (%)	444 (97.8)	*163 (36.7)*	*NS*
Mutated (%)	10 (2.2)	*4 (40.0)*	
AKT1[d]			
Non-mutated (%)	442 (96.7)	*164 (37.1)*	*NS*
Mutated (%)	15 (3.3)	*5 (33.3)*	
All mutations[d]			
Non-mutated (%)	280 (61.7)	*113 (40.4)*	***0.016***
Mutated (%)	174 (38.3)	*54 (31.0)*	

[a]Log-rank Test. NS: not significant.

[b]Data available in 456 samples.

[c]Data available in 454 samples.

[d]Data available in 457 samples
