# Supplementary material for: PIK3R1 underexpression is an independent prognostic marker in breast cancer
Source: BMC Cancer. 2013 Nov 14;13:545. doi: 10.1186/1471-2407-13-545 (PMC4225603; doi:10.1186/1471-2407-13-545)
Supplement: Additional file 5: Table S5 — Characteristics of the 458 primary breast tumors correlated with PIK3R1 expression status. [file 1471-2407-13-545-S5.pdf]

Additional Table 5. Characteristics of the 458 primary breast tumors correlated with *PIK3R1* expression status.

	Total population (%)	Number of patients (%)		*P-value*[a]
		PIK3R1 underexpression	*PIK3R1* non-underexpression	
Total	*458 (100.0)*	283 (61.8)	175 (38.2)	
Age				
≤50	*99 (21.6)*	61 (61.6)	38 (38.4)	*NS*
>50	*359 (78.4)*	222 (61.8)	137 (38.2)	
SBR histological grade[b,c]				
I	*58 (12.6)*	23 (39.7)	35 (60.3)	
II	*230 (50.2)*	125 (54.3)	105 (45.7)	***<0.0000001***
III	*161 (35.2)*	127 (78.9)	34 (21.1)	
Lymph node status [d]				
0	*120 (26.2)*	82 (68.3)	38 (31.7)	
1-3	*237 (51.7)*	131 (55.3)	106 (44.7)	***0.013***
>3	*100 (21.8)*	69 (69.0)	31 (31.0)	
Macroscopic tumor size [e]				
≤25mm	*223 (48.7)*	139 (62.3)	84 (37.7)	*NS*
>25mm	*227 (49.6)*	140 (61.7)	87 (38.3)	
ERα status				
Negative	*119 (26.0)*	103 (86.6)	16 (13.4)	***<0.0000001***
Positive	*339 (74.0)*	180 (53.1)	159 (46.9)	
PR status				
Negative	*195 (42.6)*	156 (80.0)	39 (20.0)	***<0.0000001***
Positive	*263 (57.4)*	127 (48.3)	136 (51.7)	
ERBB2 status				
Negative	*359 (78.4)*	211 (58.8)	148 (41.2)	***0.011***
Positive	*99 (21.6)*	72 (72.7)	27 (27.3)	
Molecular subtypes				
HR- ERBB2-	*69 (15.1)*	61 (88.4)	8 (11.6)	
HR- ERBB2+	*45 (9.8)*	40 (88.9)	5 (11.1)	***<0.0000001***
HR+ ERBB2-	*290 (63.3)*	150 (51.7)	140 (48.3)	
HR+ ERBB2+	*54 (11.8)*	32 (59.3)	22 (40.7)	
Patients treatments [f]				
No adjuvant treatment	*85 (19.3)*	58 (68.2)	27 (31.8)	
Chemotherapy	*89 (20.2)*	64 (71.9)	25 (28.1)	***0.026***
Hormonotherapy	*173 (39.3)*	102 (59.0)	71 (41.0)	
Chemo and Hormonotherapy	*93 (21.1)*	49 (52.7)	44 (47.3)	
		10	8	

[a]Chi^2 test. NS: not significant.

[b]Scarff-Bloom-Richardson classification.

[c]Information available for 449 patients.

[d]Information available for 457 patients.

[e]Information available for 450 patients.

[f]Information available for 440 patients.
